# Beep4Me: Automatic Ticket Validation to Support Fare Clearing and Service Planning

**DOI:** 10.3390/s22041543

**Published:** 2022-02-17

**Authors:** Giovanni Tuveri, Marco Garau, Eleonora Sottile, Lucia Pintor, Luigi Atzori, Italo Meloni

**Affiliations:** 1 Research Centre for Mobility Modelling (CRiMM), University of Cagliari, 09124 Cagliari, Italy; esottile@unica.it (E.S.); imeloni@unica.it (I.M.); 2 Electrical and Electronical Engineering Department (DIEE), University of Cagliari, 09123 Cagliari, Italy; m.garau@gmail.com (M.G.); lucia.pintor@unica.it (L.P.); l.atzori@diee.unica.it (L.A.)

**Keywords:** Bluetooth, GPS, public transport, smart ticketing, sustainability, fare clearing

## Abstract

An integrated transport service fare system, supported by an agreement for ticket revenue sharing among service providers, is an essential component to improve the experience of the users who can find single tickets for the integrated transport services they look for. A challenge is to find a model to share the revenue which all providers agree on. A solution is to adopt data-driven approaches where user-generated data are collected to extract information on the extent each transport service was used. This is consistently used. However, it suffers from incomplete data, as not all users always validate their ticket when checking out or when switching lines. We studied all technologies available to support automatic ticket validation in order to record when the users access and exit each service line. The contributions of this work are the following: we give an in-depth description of the inner workings of this novel approach describing how we take advantage of each technology; we present the developed solution (Beep4Me), which adds new functionalities to an existing mobile ticketing platform; and we describe our testing framework, which includes most cases users might encounter during a trip. Our results demonstrate how it is possible to collect key data related to validations which can be used first for clearing purposes and then for network planning/fleet optimization.

## 1. Introduction

Urban road networks are increasingly suffering from frequent congestion, making it difficult to access urban centers and causing a strong environmental impact due to CO_2_ emissions. Improving the mobility of citizens can also be achieved through the creation of an integrated fare system. It can be fundamental in triggering a modal shift towards public transport, which can in turn generate an increase in the number of available sustainable modes [1]. However, the resulting solution needs to be friendly to the users and fair for the public transport providers, who have to coexist and often cooperate with each other, especially in an urban environment. From the point of view of the travelers using public transport, the level of service offered from the whole system has to be quite high, regardless of the number of operators. In fact, since the users need to reach their destination (or destinations) from a defined starting point, for public transport to be competitive with other means of transport (mainly private vehicles), the best options available to them need to be those which allow them to finish their trip in a reasonable amount of time and with the lowest amount of effort. For this purpose, the ideal solution would be that of creating a perfectly integrated public transport system in which the providers work together to create an almost seamless network, both in the spatial dimension (e.g., the bus stops/stations are the same for every company or they are very close to each other) and in the time one (e.g., users’ waiting times when transferring from a vehicle to the next one should be as low as possible). This system should also include a unique ticketing framework, allowing users to use the same ticket everywhere on the public transport network, regardless of the vehicle on which they are traveling (bus, train, tram, etc.) and of the company providing this service. However, this also means that service providers in particular need valid and efficient methods to collect data regarding the number of passengers served with the same ticket by each of them, thus allowing them to implement the best strategies of measuring how ticket revenue has to be split.

As a matter of fact, public administrations are often concerned with finding the right way to achieve an accurate fare allocation system, and this usually leads to the institution of clearing centers [2]. This issue rises from the fact that, in a public transport system where many service providers sell fare values accepted by multiple operators, each organization must be reimbursed for the services they provided regardless of who sold the fare [3]. Difficulties in finding a shared agreement on revenue sharing can lead to dissatisfactions with transit operators who feel they are not being compensated adequately for their effective traffic volume [4]. As an example, the government of the Lazio region (Italy), produced a detailed series of guidelines, explaining how the revenue sharing models to be used in their territory is to be based on principles of equity, clarity, transparency, scientific methods, and cost effectiveness [5].

It should be noted that the main public transport providers of Sardinia were involved in all phases of our project. All of them agreed on the fact that a good clearing system is fundamental if they need to create an integrated network of different transport services. However, the current system often creates dissatisfaction among most of them, mainly since the data used can be lacking or contradictory. Thus, an improved framework would convince them to invest more resources on integrated solutions. They were also eager to see our results since the system seemed very interesting, and they would have liked to propose it to their passengers. Their willingness to improve their systems is overall a positive aspect, which would ultimately lead to them producing better services, benefitting the whole population.

Passenger data can be obtained either through systematic surveys (pricey and often less precise with respect to the adoption of automatic tools) or by taking advantage of data registered by the companies’ ETS (electronic ticketing system) [6]. Given the increasing widespread usage of personal devices smartphones to almost any age of transport service users and the growing supply of sensors/functions of these, mobile ticketing solutions should also be considered as a valid tool to collect such data and to promote a mind shift from the users to leave the private means for public transport. Such a change in citizens’ habits is also not easy since the use of public transport systems is often perceived as complex and there is a lack of seamless travel options [7]. 

Also, data collected for the fare clearing can be seen as a very precious resource, since they represent a valid tool for estimating real-time occupancy of vehicles (and consequently of lines). This would in turn allow public transport operators to better manage their fleet, distributing their vehicles in a way that accommodates the needs of the travelers. As a result, this would create a better and more efficient transport system, with obvious repercussions on the satisfaction of the users, which would then tend to use public transport more frequently and increase in number with new users attracted by an improved public transport service. 

This paper tries to find a solution for ticket validation data gathering, while also giving some kind of incentive to public transport users in order to ensure their collaboration. For this purpose, we developed a prototype smartphone application capable of using several systems in order to identify a vehicle (bus, tram, or train) and consequently validate a ticket in a completely automated way. This paper is an extension of our previous work [8], in which we present the results we achieved after completing the system with all of the functionalities we wished to implement, and integrating it in the already existing mobile ticketing app. Also, we could test the system by installing our Bluetooth low energy (BLE) beacons on real buses instead of resorting to using temporary solutions (e.g., placing the beacons inside a moving car) as we did on our first tests.

The main contributions of the paper are as follows:

- We define an innovative approach based on the simultaneous use of Bluetooth low energy beacons and of different sensors (Bluetooth, location and orientation) commonly found on all smartphones.
- We present a new integrated solution we developed called Beep4Me, which completes a pre-existing mobile ticketing platform (Busfinder [9]) with the possibility for the users to activate the automatic validation option, giving them a more comfortable and seamless ride experience.
- We show the analysis of our field tests, which aimed to try-out the system in almost every possible circumstance the users might encounter during their trips, and verify the new framework worked correctly in each one of them. The results from these tests confirm the efficiency and stability of the system.

The remainder of the paper is organized as follows. Section 2. gives a picture of what has already been studied and tested in the field of more or less automated validation systems. In Section 3. we present our proposed solution (Beep4Me) and the technologies it uses to achieve the automatic validation. Section 4. gives instead a more in-depth description of the inner workings of the Beep4Me system, explaining how each technology can be used in every step of the process. In Section 5. we describe all of the various tests we performed and show the results of our experimentation. Finally, Section 6. presents the more significant findings of our work and the actions we plan to take to develop the system even further.

## 2. Past Works

With reference to ticket validation, there are mostly four different schemes of user interactions when entering and leaving a transport vehicle [10]:

- Check-In only (CI)—users only have to act when boarding a vehicle.
- Check-In Check-Out (CICO)—users have to act both when boarding and when alighting.
- Check-In Be-Out (CIBO)—users only have to act when boarding, their alighting is automatically detected.
- Be-In Be-Out (BIBO)—the presence of the users is detected automatically during both boarding and alighting, so that no action is required.

The CI alternative is probably still the most widespread, given the simple implementation compatible even with basic paper tickets, while other systems require specific hardware on the vehicles or/and in the stations. The automation provided by CIBO and BIBO could allow to build a complete and reliable database by continuously gathering validation data; however, the number of successful real-world applications is still limited. A BIBO scheme, which drastically reduces the uncertainty due to user actions in the recording of trips data, could prove to be one of the best technological applications currently available for improving public transportation as a whole. Table 1 presents a summary of the pros and cons of each of these validation schemes.

One of the first BIBO proof of concepts, which used standard Bluetooth, was presented by Ericsson in 2001. In 2004, in Switzerland, “ATRON” showcased a CIBO prototype, which used a WLAN antenna [10]. In 2007, the Swiss Federal Railways issued a “Request for information” to the industry, to identify a BIBO solution for all national public transport [10], which led to the development of two CICO smartphone apps [11,12].

The first BIBO documented large scale tests come from the “ALLFA” project, developed by Siemens in 2005 and tested in Dresden. The users received an electronic device, either a “smart card” or a mobile phone, whose signal was automatically detected by antennas mounted inside the vehicles [10].

An Android app was tested in 2014 at the Norwegian University of Science and Technology to automate ticket validation at events by installing BLE (Bluetooth low energy) beacons at the various venues [13]. In 2016, software engineers at Johannes Kepler University tried to prove whether BLE was suitable for public transport BIBO systems [14].

In 2017, a BIBO concept app, which interacted with BLE beacons, was developed in the Netherlands and was tested in a makeshift mini-bus by 20 test users [15]. In the same year, a mobile ticketing app which used the BLE technology was developed for Porto’s intermodal public transport network [16]. However, the system was converted to a CIBO layout, keeping BLE for the BO operations while using NFC (near field communication) for check-in [17].

A system developed by the company “Turnit” was more recently used in the Estonian city of Tartu. The system automatically detected passengers boarding and alighting thanks to BLE beacons installed on the buses [18]. During the testing phases, about 2500 testers completed over 60,000 trips using Turnit BIBO. However, the system was not completely successful, and testing has since been stopped. Feedback from the testers showed that a BIBO system is both welcome and convenient [19].

The earlier experiences relied on a single technology, either Wi-Fi or Bluetooth, whereas current approaches often use more than one technology, each one used to address the disadvantages/the shortcomings of the others [7]. Our system, which can work as both a CIBO and a BIBO system, can use different technologies at the same time to validate the travel ticket: QR (Quick Response) codes, BLE, GPS, and data from the smartphone’s motion and orientation sensors. It also uses 3G/4G connection to send useful data to the servers of the transport service provider. The choice of the ticket validation method is ultimately left to the users, who will decide with which configuration they feel more comfortable. Also, many of the previous experimentations included some sort of system to automatically pay for tickets. However, the prototype we are proposing focuses only on a system that automates ticket validation, relying on an existing system for the purchase of travel tickets and prompting users to buy a new one only when they don’t already have one available. We leave the possibility of including automated ticket purchase in a future version of the prototype.

## 3. Overview of the Beep4Me Solution

Beep4Me’s name reflects the purpose of the system, which ultimately is that of executing all validation operations for the users. Since the validation confirmation on public transport vehicles is usually accompanied by an acoustic signal (“beep”) the system was given the name “beep for me”, meaning “validate for me”. Beep4Me is a BIBO functionality that relies on an existing mobile ticketing solution that allows for buying digital tickets and using them whenever needed. The assumption is that existing system relies on CI/CICO modality for ticket validation, which may be based on either: (i) an NFC system in the vehicle to read the tickets; or (ii) QR codes located in the walls of the vehicles and that have to be scanned by the smartphone camera to validate the tickets. In the following we assume that the scenario (ii) is the one currently deployed, as this is the one that characterizes the on-field experiments. However, it does not prevent us from implementing Beep4Me in scenario (i). The following sections describe the users’ interaction and the adopted technologies. 

### 3.1. User Interaction

**Figure 1:**
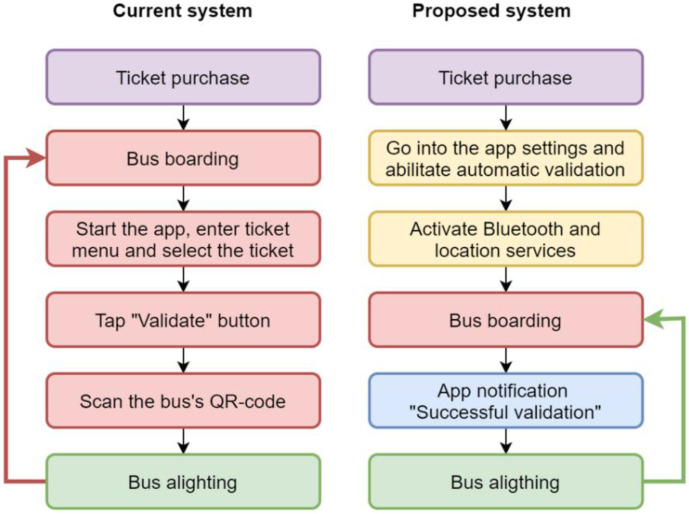
Flow diagrams for user actions before (**left**) and after (**right**) the introduction of automatic validation.

Figure 1 shows the different sets of actions that any user must carry out when using the smart ticketing app, both currently and when the proposed system will become operational. We assume that the users, whenever they want to travel using public transport, must have a valid ticket, purchased on the app, which they must then validate for their ride. The left side of the diagram in the figure shows the sequence of operations required to use the current system, while the right one indicates the set of different actions needed when the automatic features become available.

Specifically, in the current configuration, users, after boarding a bus, must open the app, access the tickets menu, select the desired ticket, start the validation process by pressing the “Validate” button, scan one of the QR codes placed on the vehicle, and finally get off the bus when they arrive at their destination. The same steps must always be repeated every time they board another bus, from opening the app to scanning the QR code.

Whenever the new proposed automatic validation features will become available to the passengers, the users who want to use PT (public transport) will have to follow some steps similar to those of the current system. First, after purchasing a ticket, the users will have to enter the tickets menu of the app and manually activate the automatic validation option. Then they will have to make sure their Bluetooth and/or the location services on their devices are also turned on, since the system will not work without at least one of them being active. They will also have to make sure these systems remain active as long as they wish to use automatic validation (this is a smaller issue, considering how often people keep at least Bluetooth always on, given the large diffusion of wearable devices such as smart watches and audio devices). To affect battery life as little as possible, smartphones will communicate with the backend/server system only when necessary.

All previously described steps have to be taken only once, unless the users wish to stop using the automated system or start using it again after having deactivated it. Henceforth, the users can have a completely seamless travel experience, since if they have a valid ticket, it will be validated even if the phone is in their pocket. If the users are using the mobile phone, their activity will not be interrupted, unlike what happens for example with mobile ticketing systems based on NFC [7].

In fact, after boarding a vehicle, Beep4Me will then automatically send a pop-up notification to the users, informing them their ticket was successfully validated. They will then be able to travel on public transport without any other active interaction for as long as their ticket is valid, boarding and alighting from as many vehicles as they wish and only receiving the occasional notification.

In the context where we conducted our experiments, most tickets that involve multiple service providers are available only through the mobile app. This fact implies that passengers are forced to use the app if they want to use this kind of ticket, meaning the revenue sharing issue is only affected by smartphone users. However, all non-mobile tickets are hidden from our system in other contexts, and companies should have an alternative clearing system reserved for them. Nevertheless, our system would allow companies to have at least a valid point of reference, which could be also used to extrapolate shares by considering a combination of the validation data from Beep4Me and the percentage of users who use Beep4Me.

### 3.2. Used Technologies

The new app-based system will expand the current one (i.e., BusFinder and ATP Sassari), Beep4Me allows for validating the mobile tickets adding the option of using it as a BIBO system, giving the users a completely hands-free experience. This result is achieved by using, at the same time, the Bluetooth, the motion sensors, and the location services, all available on most smartphones, while keeping the current QRcode system. These services can work independently and in combination, by taking into consideration each user’s smartphone configuration. Most of the used technologies are already well-known:

- QR codes usually consist of black patterns arranged in a square grid on a white background, which can be read by a camera and processed to extract the data they contain [20].
- The smartphones’ location services allow apps to access the data gathered from the satellite system (GPS, GLONASS) by dedicated antennas to determine the geographical coordinates of the smartphone [21,22].
- The motion sensors (accelerometer and gyroscope) installed in most smartphones register relative accelerations on three axes and orientation and allow, through specific algorithms, to determine the kind of user’s motion activity [23,24].
- Bluetooth is a wireless technology standard for data exchange over short distances using radio waves in the 2.4 GHz band [25]. Bluetooth low energy (BLE) is an evolution of the standard, requiring considerably lower power (years of autonomy achievable using a single button cell) with a small size and low cost [26].

A BLE beacon is a device that broadcasts a series of identifiers, enabling receiving devices to perform various actions. Beacons can operate by using different protocols [27], among which iBeacon by Apple Inc. is the most successful. An iBeacon information advertising provides a UUID (Universally Unique IDentifier) (16 bytes), a Major (2 bytes) and a Minor (2 bytes), which can be customized to fit any possible application [23]. In addition to these values, when advertising an iBeacon will also return a value for “accuracy”, the accuracy of the proximity in meters from the beacon (different from the actual distance from the beacon) and one for “RSSI” (received signal strngth indicator), the average value of the power level samples received from the beacon [28]. Since the “accuracy” returned by the beacon is influenced significantly by how each beacon is calibrated and by the presence of physical obstacles between the beacon itself and the devices receiving the broadcast, we chose not to use these values which often not accurate at all.

**Figure 2:**
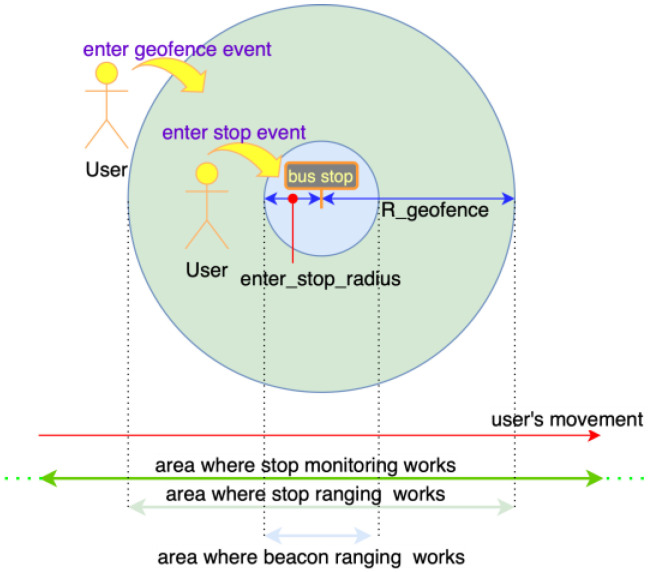
Detecting enter stop.

**Figure 3:**
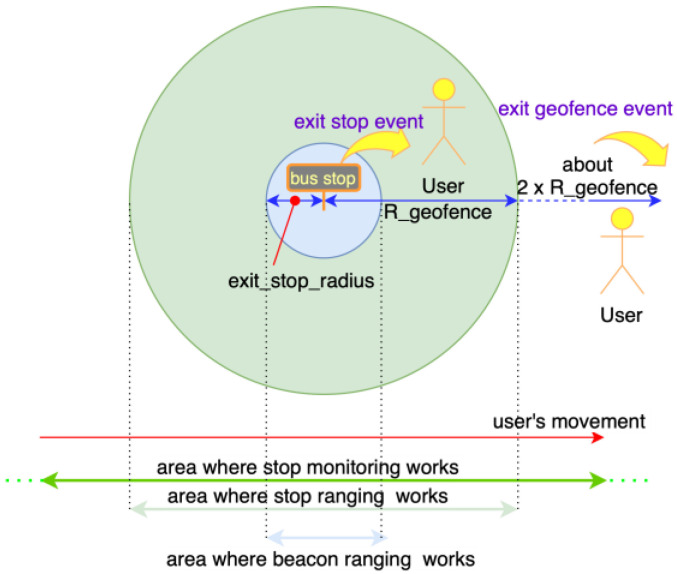
Detecting exit stop.

When approaching certain designated physical locations, smartphone applications can react mainly in two ways:

- Region Monitoring—this system allows the application to understand when a device enters or exits a so-called “region”. A region can be defined by either:
  1. A UUID associated with an in-range BLE beacon physically installed in such location.
  2. A radius (R_geofence) and a center (a geographical position) which define a circular region, called geofence. In the case of Beep4Me, the center is set by using the coordinates of the nearby stops for public transport companies, while R_geofence is set on a default value (shown in Table 2).
- Ranging—when the system enters a region, it returns an approximative level of distance (immediate, near, far or unknown) and a received power level from all of the beacons therein [28] and a quite accurate value of distance from the center of the geofence (Figure 2 and Figure 3).
- Region monitoring and ranging technologies will be more thoroughly described in Section 4.1. While location and motion sensors functionalities do not require any additional hardware, BLE requires beacons to be installed inside the vehicles. During a testing phase a wide set of options is preferred, so we choose to use BLE beacons providing a good level of customization (datasheets detail are available in Appendix A) [29]. As of now, only the iBeacon protocol has been implemented during testing, using the UUID as the system unique identifier, the ‘Major’ as the bus and company identifier and finally the ‘Minor’ as the beacon identifier inside the vehicle.

## 4. The Beep4Me Ticket Validation Algorithm

### 4.1. Beep4Me Workflow

After updating the app with the Beep4Me system, the new functionalities which allow to simplify ticket validation procedures, usually executed while traveling on public transport, will be presented to users by means of a pop-up window. Users will receive the option to enable the new automatic validation functionalities by activating a switch in the app, which they will find in their ticket wallet. The app-side user experience is based completely on the use of push notifications and banners giving them updates on their trips and on the status (i.e., validation) of their tickets. These notifications provide them with a visual confirmation of the check-in and check-out events, and thus of the correct operation of the whole system, while requiring a minimal amount of actions to successfully perform a validation.

**Figure 4:**
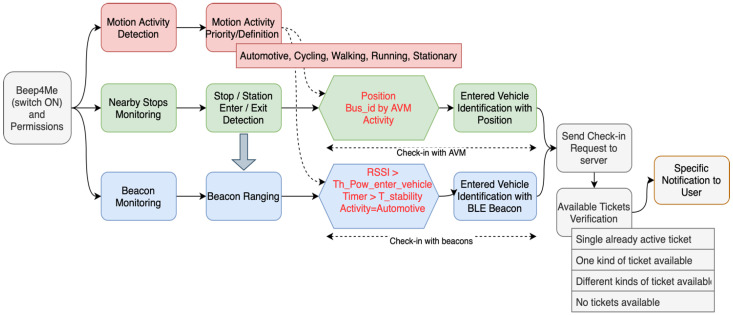
Flow diagram of the automatic check-in operations.

Figure 4 shows the workflow of the algorithm from the activation of the Beep4Me functionalities up to the moment in which the check-in operations have been completed. The algorithm is based on sensing operations aimed at determining the presence of a user inside a public transport vehicle (among those present in the system’s database).

On activating Beep4Me through the switch (first block in Figure 4), the application will present users with a dialog asking for confirmation they wish to give all necessary permissions and to activate the needed sensors (Bluetooth in particular) for using the new functionalities, always giving them the option to choose whether to accept or not. If they do not agree to these terms, Beep4Me will not be used.

These operations have the final aim of reducing the probability of false positives, which might happen when one of the vehicles travels by or stops by users located outside of the same vehicles (e.g., since they are walking nearby or they are waiting inside a bus-stop region for another vehicle/bus-line).

The detection of motion activity (red blocks in Figure 4) uses several smartphone sensors: accelerometer, gyroscope, and magnetometer, when available. It is a service which estimates the activity of users in motion, choosing between the different available activities (i.e., walking, running, stationary, cycling, and automotive, (“Motion Activity Priority/Decision” block)). Motion activity detection is always active when Beep4Me is working, since it is a sensing technology which uses very low amounts of power, but more importantly since the other sensing systems used need to have a value for the motion activity as much updated as possible in order to determine the presence of users on a vehicle (as explained later). 

Since the estimation of motion activity is associated with a certain confidence level (low, medium, high), choosing between the detectable activities is sometimes trickier for the system and might be slower. This can happen, for example, when users are changing their activity from walking to stationary and then finally to automotive. In order to make the choice more immediate, having it happen right after activating the sensing of motion activity, we defined a priority setting based on which the app will have to decide between the activities. 

This was also revealed to be a necessary adjustment during the experimentation on public transport vehicles (more details on this can be found in Section 4.2). Once the user activity has been successfully identified, it is recorded and frequently updated (once per second during regular operations), and it is then used for the other two sensing systems of Beep4Me. 

Motion activity data is in fact useful when determining the presence on board of the users through geolocation and beacons, especially to understand if the enter and exit events (regarding the regions) are actually significative for the automatic check-in process (and also for automatic check-out, as described later), as can be deduced from the red-text blocks in in Figure 4.

Geographic region monitoring, associated with the closest bus stops and stations, is necessary to determine the presence of the users inside the areas of the same public transport stops. Figure 2 and Figure 3 show the processes which were implemented for analyzing the various phases of entering in and exiting from the regions of the stops.

It is possible to detect the access in (enter_geofence_event) and the egress from (exit_geofence_event) a geofence by using a smartphone, with a function managed by the operative system (OS) itself, which uses low amounts of power.

For this purpose, several technologies are used jointly: the geofence system and user location data; information obtained through the AVM (Automatic Vehicle Monitoring) system of public transport operators; the iBeacon framework; smartphone motion sensing. Thus, the system has been developed in such a way such that it activates a check-in correspondence of the following events:

- Check-in with beacons: users have been at a stop/station, they had an “automotive” status at least once and have been in a beacon-covered area for a certain length of time.
- Check-in with AVM: users have been at a stop/station, they had an “automotive” status at least once and their position overlapped with that of a vehicle for a definite amount of time, going by what indicated by the AVM system.

At this end, once it starts, the system performs in parallel three types of sensing: (1) motion activity detection, (2) close-by stops regions monitoring, and (3) beacon regions monitoring. More details on all of the carried-out operations are given in the following paragraphs.

The minimum available value for R_geofence is although too high (Table 2) and thus it does not allow for an accurate detection of the users in proximity to the stops, so it is necessary to perform a ranging of the stops using the current position of the users. Ranging is a more energy intensive process when compared to monitoring, but it allows to determine the ingress/egress of the users from an inner smaller zone of the bus stops, comparing the distance estimated through ranging with a radius (enter_stop_radius) smaller than R_geofence (light blue area in Figure 2). We decided then to use both these types of sensing to reach a compromise between a sufficient precision and a non-excessive expenditure of smartphone resources when determining the presence of users inside the circular area of the stops of radius enter_stop_radius.

This data also influences the flow in the sensing of the beacons: in particular, as seen in Figure 2 and Figure 3, the enter (or exit) event in the stop area (light blue portion in Figure 2) influences the activation of the ranging of beacons (blue “Beacon Ranging” block in Figure 4) by either allowing or blocking it. This is useful for avoiding check-in events outside of bus-stop areas, which would presumably be wrong, for example due to the users standing close to one of the beacon-equipped buses (which led to some problems during experimentation, see Section 4.2).

Determining the presence of users inside the area of radius enter_stop_radius, allows to record the events enter_stop_event (Figure 2) ed exit_stop_event (Figure 3). If these events are associated to a detected activity of category Walking and Automotive, respectively, the system classifies them as “presumed start/end of the trip” and queries the dedicated API (application programming interface) of the AVM system of the public transport providers, to receive information about any vehicle potentially being in the proximity of the bus stop when the event occurred. By correlating all this information, Beep4Me can determine the presence of users on board of the vehicle identified through the AVM system (last two green blocks in Figure 4). At this point, the system sends a check-in request to the Beep4Me API of the public transport company ticketing server.

In parallel to geofence monitoring and motion activity detection, Beep4Me starts the beacon region monitoring, which begins by defining the beacon regions to be monitored. It basically consists in the definition in app of the beacon groups to which the app itself has to react, that is which are the beacons the app has to “listen” to between all of the beacons which might be in proximity to the smartphone. Physically speaking, the beacon regions are the areas covered by the signal of the beacon group whose characteristics reflect those defined for that region. There are mainly three ways to define beacon regions using different combinations of the iBeacon protocol codes, which are UUID, *major* and *minor*.

All of the beacons belonging to the system have an UUID defined by the beacon region, while *major* and *minor* get used to differentiate the vehicle (and the company) and the beacon inside the vehicle, respectively. Specifically, when the OS (monitoring function) detects the access inside a beacon region (the smartphone physically enters the area covered by one or more beacons in that region) it also activates the Beep4Me ranging if the users are located inside a bus stop area, as previously explained. The ranging system estimates the proximity (a value from immediate, near and far, intuitive but not reliable), the distance from the beacon (again, not reliable) and the power (RSSI dBm) of the signal received by each of the beacons. This information is extracted by the smartphone from each advertising packet received, which consists of an information package broadcasted by the beacon with a frequency interval of 1/(Advertising interval) seconds.

**Figure 5:**
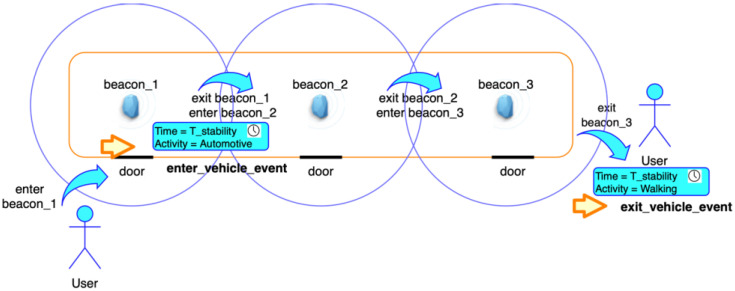
Diagram showing the interactions with a bus equipped with three beacons.

In this way, it is possible to understand if users are close enough to the beacons (belonging to the beacon region) having a certain *major* value and thus identifying a certain vehicle of a public transport company. If the power value (RSSI dBm) received from one or more beacons installed on a given vehicle (effectively identifying it) exceeds a predetermined threshold Th_Pow_Enter_Vehicle dBm (see Table 2) for a period of time longer than T_stability seconds (Table 2) and the current motion activity is Automotive (blue block with red text in Figure 4), then the system considers the users to be on board of the vehicle identified by his *major* (blue “Entered Vehicle Identification with BLE Beacon” block in Figure 4). The power threshold Th_Pow_enter_bus dBm depends on the environment in which the system has to function (mainly the type of vehicle) and the use-case and it has been set on the value present in Table 2 for identifying the enter_beacon event (see enter_beacon_1 in Figure 5). In Figure 5 we highlight the distinction between the enter_beacon event, caused simply by the RSSI from beacon_1 going over the Th_Pow_enter_vehicle threshold, ant the enter_vehicle event, caused by the RSSI going over the same threshold but for continuous period of time of T_stability seconds and by the measured motion activity being equal to Automotive. The power threshold depends also on the transmission power level Beacon_transmission_power dBm (Table 2) set for the beacons installed in the vehicle. The time interval T_stability is also dependent on the use-case and the working environment for the system. As an example, in all of the cases which require a higher level of accuracy, and an extreme system reaction speed when validating tickets is not needed, T_stability can be increased to also increase the level of confidence of the system when detecting the enter_vehicle event (Figure 5). The values set for per Th_Pow_Enter_Vehicle dBm and T_stability seconds in Table 2 reflect the experimental setup (described in Section 5.2) used during the experiments carried out between July and November 2020.

Once the presence of users on-board the vehicle is ascertained, whether the vehicle ID was obtained through the AVM system or using the *major* of the installed beacons (when present), the app sends a check-in request to the Beep4Me API integrated in the PT provider ticketing server. The server performs a check on the users’ tickets and distinguishes between four different cases when users board a vehicle, each associated with a corresponding user notification. Based on the scenario, a different behavior is activated:

- If the users own one or more tickets, all of the same type and not yet validated, the system proceeds to the validation operations and answers to the app with a successful automatic validation notification, “Congratulations! The *12 xyz* ticket was automatically validated on *21 February 2021* at *12:34 P.M*.” Clicking on this notification, users can see the just validated ticket and a note informing them that the validation happened automatically.
- If the users have an already validated non-yet-expired ticket, the system proceeds to perform a transfer operation and answers to the app with a successful automatic transfer notification, “Congratulations! You completed a transfer with the *12 xyz* ticket on *21 February 2021* at *12:34 P.M*.” Clicking on this notification, users can see their ticket and a note informing them that the operation happened automatically.
- If the users possess more tickets, of different types and not yet validated, the system sends a specific notification, asking the users to select one of them “Warning! Several tickets available! Please choose the one you wish to validate.” Clicking on the notification, users will be led to a selection screen where a list of all available tickets will be shown. The chosen ticket will have to be validated by simply using a “Validate” button, which will end the validation process without needing to scan any of the vehicle QR code.
- If the users possess no tickets whatsoever in their wallet, the system will show a notification informing them of this issue, “Warning! No tickets available. Please purchase one and validate it.” Clicking on this notification, users will immediately reach the “Ticket Purchase” section of the app, where they will have to choose one and buy it. Once a ticket is purchased, the validation can happen manually, or it will automatically happen after waiting a few seconds, once the system has recognized the presence of the newly-bought ticket, as in the first case where only one ticket was available (given that the users are in proximity to the beacons and all of the conditions for the automatic validation process are valid).

**Figure 6:**
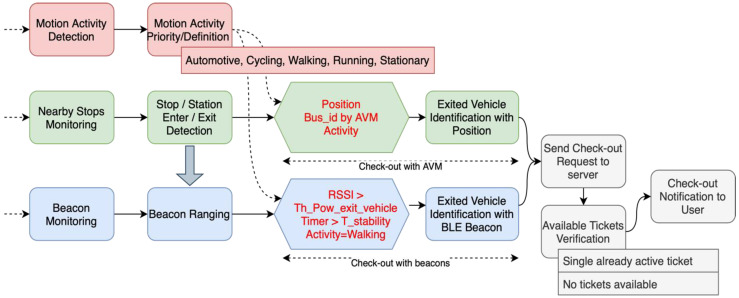
Flow diagram—check-out operations.

Figure 6 shows the workflow happening in the app when the check-out operations are finalized. Using the sensing in Beep4Me in this case is needed to detect the alighting of users from the vehicle which they previously boarded. The boarding and the vehicle ID have been already identified during the automatic check-in process, and the same technologies can be used to identify the alighting. As can be noticed in Figure 6, we presume Beep4Me to be already active (i.e., the switch is ON), a check-in already happened, and the trip is on-going, that is the users possess a non-expired validated ticket in their wallet and the smartphone sensors are notifying the system that the users are still on board a vehicle.

As pointed out in the three left-most blocks in Figure 6, the flow can be described starting from the processes used for determining motion activity, geofence sensing, and beacon sensing, which are active and detecting the presence of the users on the vehicle. Motion activity detection works exactly such as in the case of check-in:

- A priority is set between the possible activities based on the difficulty of detection (more details in Section 4.2) and on their importance for the correct functioning of the algorithm (in order, automotive, cycling, walking, running, stationary).
- For each of these activities, the system estimates a probability and chooses the most probable one as the one the users are performing.
- The system memorizes this activity and updates it frequently, to make it available to the other sensing processes (position and beacons). When these processes need the activity to confirm the results of region ingress/egress events, they pick the current value from the motion detection output (dashed connections from the second red block in Figure 6).

Geofence monitoring for check-out is necessary for defining the stop region ingress/egress events, while associating them to a particular type of motion activity. In the same way it was used for check-in, the detection of the enter in/exit from a stop region through geofence technology happens for a distance from the stop which is usually too large, so using the ranging of the stops was needed also in this case. Comparing the distance from the stop obtained by ranging with the values of the radii enter_stop_radius and exit_stop_radius (Section 5.2.), the enter_stop and exit_stop events in the internal area of the bus stop/station are determined exactly (blue area in Figure 3 and green “Stop/Station Enter/Exit detection” block in Figure 6). If the enter_stop and exit_stop events are associated with an Automotive and Walking (or Running or Stationary) activity, respectively, the system classifies them as “presumed end of the trip” and queries the provider AVM system to obtain information regarding possible vehicles which recently passed by the same stop. At this point, the system sends a check-out request to the Beep4Me API integrated in the PT provider ticketing server (grey “Send Check-out Request to server” block in Figure 6). 

To describe the automatic check-out process when using beacons, we considered the starting circumstance in which beacon sensing is already active, the user is on the vehicle, and the system is already detecting the beacons installed on that vehicle. The system must have recorded RSSI powers higher than the threshold value Th_Pow_enter_vehicle dBm for a time longer than T_stability seconds (see Table 2 for the values used during the tests), that is an enter_vehicle event that must have already happened (Figure 5) and a validated (either by check-in or transfer) ticket is present in the users’ wallet. Once the users have alighted from a vehicle (and get further away from it), the RSSI powers from the advertising packets should be showing decreasing values over time. If these values are maintained under the threshold Th_Pow_exit_vehicle dBm (Table 2), for a time longer than T_stability seconds and the current motion activity is Walking, Running or Stationary (if the users are waiting for the next vehicle in the case of a transfer), then an exit_vehicle event is generated (Figure 6). This means the system has detected an egress of the users from a PT vehicle, identified by its ID obtained through the *major* of its beacons, whose signal was recently lost by the app. At this point, the system sends a check-out request to Beep4Me API integrated in the ticketing server. 

Once the back-end system receives the check-out request, regardless of the method used for identifying the vehicle (beacons or location), the server will check the users’ tickets. Two situations might happen: (1) there is an active already validated not-yet-expired ticket or (2) there are no active tickets. In the first case, the system performs all of the check-out operations and sends a response to the app, which shows a notification “Congratulations! You completed a check-out automatically with the *12 xyz* ticket on *21 February 2021* at *12:34 P.M*.” Clicking on the notification, users can see the pertinent validated ticket and a toast banner informing them that the check-out happened automatically. We would like to stress the fact that performing a check-out is not currently mandatory in the PT systems active in Sardinia. However, the check-out notification was added in the system as a verification method for checking the correct behavior of one of the features among the most interesting for PT companies: as a matter of fact, detecting the check-out events allows the providers included in an integrated public transport system to automatically measure the permanence of users on boarding of their vehicle and facilitates clearing operations. 

### 4.2. Changes to the Algorithm during Experimentation

During some tests in the month of July 2020, we encountered some technical difficulties when using older devices while identifying some of the motion activities necessary for the automatic validation. This was particularly noticeable when the vehicle was moving slowly (speeds of 30 km/h or lower), since the vibrations detected by the motion sensors were so mild in intensity that they were either ignored or delayed (by approximately 30 s) compared to the start of the movement of the vehicle. This issue was more relevant on the older smartphone (iPhone 6, 2014), while it seemed to be absent on newer models (equipped with an updated motion sensor chip).

This led to a slight modification of the algorithm, specifically to our choice of managing the priority of the different activities, assigning a higher priority to the ones which prove to be harder to detect, which was “Automotive” in our tests. This means that, when the algorithm is unsure between “Automotive” and another activity (usually “Walking” or “Stationary”) the app automatically chooses “Automotive”, and the process of requesting for a check-in can advance. The other activities, in order of priority level, are “Cycling”, “Running”, “Walking”, “Stationary”, “Unknown”. The logic is always that of increasing the accuracy of identifying the events which are harder to detect and at the same time more important, i.e., boarding a vehicle (Figure 5 enter_vehicle _event).

Another change introduced, compared to the first demo-app, is the use of geofences. Geofences are defined geographical areas identified by a specific virtual perimeter [30]. We chose circular geofences (radius is R_geofence) inside which we activate a specific monitoring of the iOS operative systems. Whatever the status of the app (this includes the “killed” status) that monitors the geofences, iOS recognizes the entrance and the exit from them, and transmits this information to the app with a specific event [31]. In our system, geofences are associated to each bus stop/station and they are used to activate the ranging of the “station”, a process which uses the users’ GPS coordinates to calculate the distance from the bus stop/station and detects when they “enter” the “station” (the distance threshold is Enter_stop_radius m, Table 2).

In this way, the system can guess the start of a trip and activate the ranging of the beacons (which will be installed in the next PT vehicle arriving at that stop). If the users leave the stop area (distance higher than Exit_stop_radius m) without coming across any beacon, the system immediately disables beacon ranging to save battery. More so, after exiting from the stop geofence, stop ranging is also disabled to save smartphone battery. This brings Beep4Me back to the status of monitoring geofences and beacons at an OS level, meaning battery optimization is as high as possible. Given the issues emphasized especially during the tests regarding “waiting at a bus stop without boarding a vehicle”, geofence technology could also be used to block ticket validation inside the stop/station areas, avoiding wrong validation in all of the cases when smartphones would incorrectly detect the activities, and allowing it only after leaving the circular area of radius Enter_stop_radius m around the stop. 

### 4.3. System Prototype

**Figure 7:**
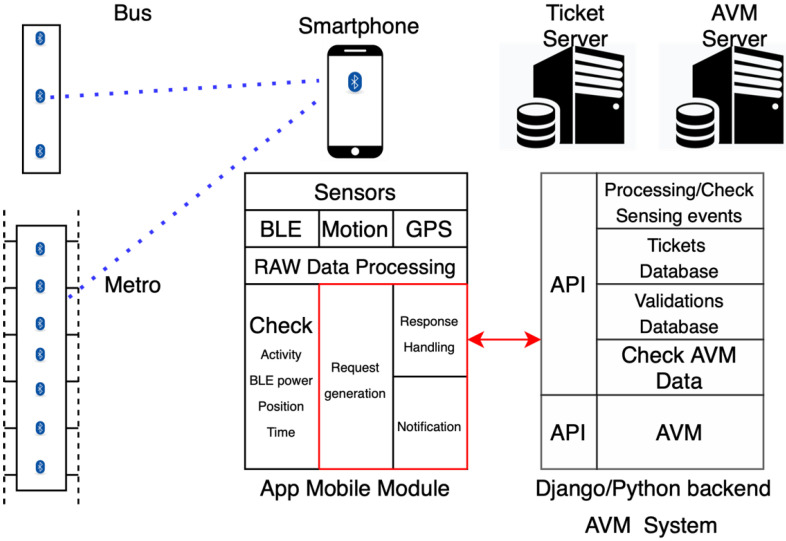
Complete system architecture.

In order to measure the permanence of a user inside a bus and to test the whole validation process based on Beep4Me, the new features have been added to an already existing app, since it is already supplied by the company CTM, which collaborated in the SIMPLE Cluster project [32]. This modified version of the app was used for online vehicle testing. Alternatively, a completely new app will be developed for the companies that do not already have one. The new system architecture is shown in Figure 7.

The Beep4Me mobile module has been developed for iOS systems in Swift language. It integrates in a 1:1 copy of the current BusFinder app (CTM), adding Beep4Me features, thus allowing all existing functions, as for example the manual validation by scanning the QR code.

The Beep4Me server module, developed in Python with the Django framework, consists of some APIs integrated into a copy of the CTM ticket validation management and control server system, allowing the exchange of important information. This information is necessary to complete the validation process and communicate to the user the actual status of his ticket, according to the scheme of notification shown above. 

## 5. On the Field Experiments

The experiments were all performed in the Metropolitan City of Cagliari (Città Metropolitana di Cagliari), by using an integration of the Beep4Me prototype on the already existing “Busfinder” system, used for info-mobility and mobile ticketing purposes by several PT providers (CTM, ARST, and Baire). We will beforehand present the first batch of tests, carried out mainly to ascertain the reliability of the employed sensors and perform a preliminary calibration. Afterwards, we will show the settings used on the on-line vehicles, the test scenarios and the results obtained. 

### 5.1. Sensors Performance Analysis and Calibration 

During the first tests, we used a demo simplified app whose primary objective was to collect data from all sensors which are going to be used in the final version (i.e., Bluetooth, motion sensors, GPS, and camera (for QR codes)). This preliminary test phase was dedicated to testing sensor operations and stability, to find the corresponding events needed to achieve the automated process of ticket validation. 

The first batch of tests focused on analyzing the accuracy in the detection of all of the phases of ticket usage on numerous different physical settings. Each of these tests was repeated five times, from start to finish using at least three beacons each time. To test a more “stressful” system configuration, some of the tests were carried out using two cars which represented two “fake-buses”, each equipped with a set of three beacons and two different QR codes. The different scenarios tested were the following:

- Detecting beacons while riding on a bus—this test was necessary to collect data in a situation as close as possible to that in which the beacons will operate when the system becomes fully operational. Since installing beacons on the vehicles was not yet possible, we operated by manually bringing the beacons on board for each test. The testing team needed one app tester, and three “beacon carriers”, who held onto a beacon each the whole time.
- Switching from a bus to another—this test was performed to examine the system behavior when a user alights at a bus stop and immediately boards another bus positioned in front/behind the previous one. To simplify the tests, and since the beacons are not influenced by the movement of the vehicle, the test was performed when the two buses were parked one behind the other.
- Detecting beacons placed on two different buses—the testers rode on a bus equipped with beacons (bus A) while, close by, another bus (bus B) is traveling at a very short distance with its own beacons. We wanted to know how the system behaved when the app detected the presence of two different sets of beacons, to calibrate them to avoid any possibility of false positives (i.e., incorrect validations on the vehicle on which the person is not riding).
- Waiting at a bus stop where a bus is idle—the testers had to wait close to a beacon-equipped bus without boarding, to simulate what usually happens at a bus terminal where several bus lines converge. In this case also, we wanted to collect data to calibrate the system to avoid incorrect validations (i.e., on one of the nearby buses the testers did not board).
- Waiting at a bus stop with no nearby buses—unlike the previous scenarios, in this case the testers had to wait at a generic bus stop and wait for a bus on which beacons were installed to pass by. Again, we aimed at collecting data to avoid false positives (i.e., tickets should not be validated due to the passage of the beacons nearby).

**Figure 8:**
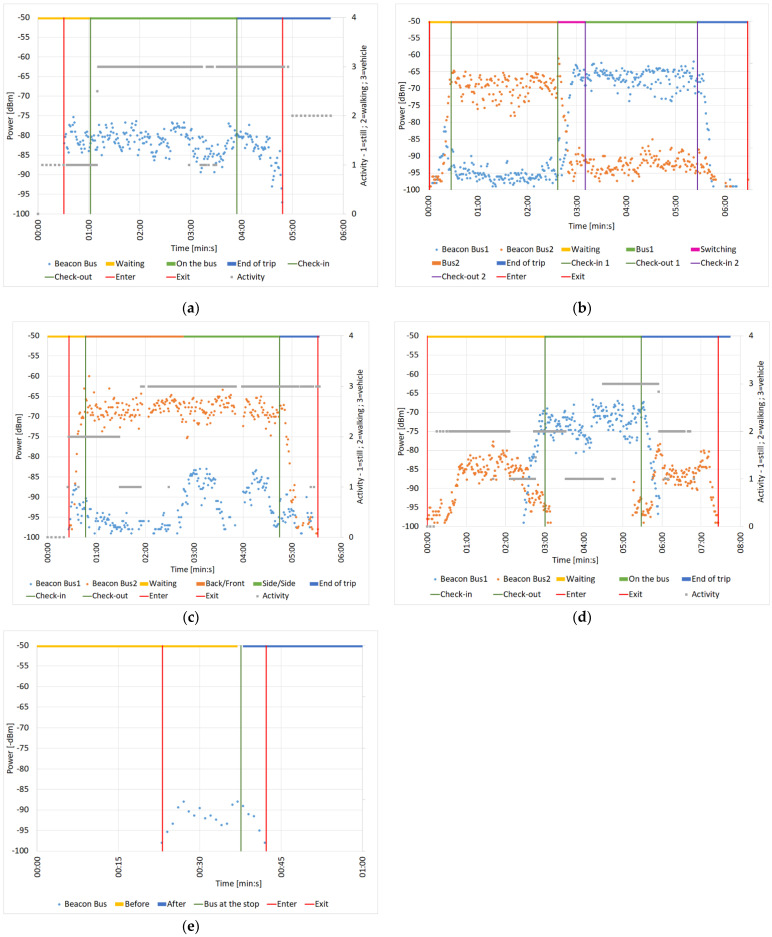
Results of the first batch of tests for different scenarios. (**a**) Detecting beacons while riding on a bus; (**b**) switching from a bus to another; (**c**) detecting beacons placed on two different buses; (**d**) waiting at a bus stop where a bus is idle; (**e**) waiting at a bus stop with no nearby buses.

Figure 8 shows some of the most significant samples of data collected during these tests, for each of the testing scenarios. In these graphs the “enter” and “exit” events from the region covered by the beacon signal are represented by two vertical red lines, while the first set of check-in and check-out events is two vertical green lines, and the second one (when present) is two vertical purple lines. The motion activity levels were represented using a numeric scale, in which 1 corresponds to “Stationary” (“still” in the legend), 2 is “Walking” or “Running” (“walking”) and finally 3 is “Automotive” (“vehicle”). 

All power levels represented are extracted from the data received by smartphones from the beacons. The RSSI is the estimated power value in dBm of the signal collected by the smartphone from each advertising packet received, which consists of an information package broadcasted by the beacon with a frequency interval of 1/(Advertising interval) seconds. 

The accuracy could be obtained through experiments in a controlled environment. Each experiment should be repeated with different source devices and the results compared. It is proven that accuracy is mostly influenced by the vertical orientation of the antenna and that it can vary up to 23 dB.

However, RSSI reception depends on the smartphone model: different devices have different sizes, casing materials, and number of antennas [33]. Furthermore, a single measurement is not sufficient for the estimation, thus these measurements are calculated through an average of at least three consecutive samples. The proximity of walls or other obstacles, for example human bodies or seats, changes the signal strength and complicates the estimation of the error [34]. It should also be noted that all observations to this graph should account for smartphones sensitivity when calculating the value of a RSSI, which can be considered in the range ± 3 dB [35].

Figure 8a shows the power values of the three beacons placed on a bus for the Detecting beacons while riding on a bus scenario. Immediately after the first red vertical line (i.e., entering the region of the first detected beacon), power values start to be detected, with an average value of −80 dBm, (with fluctuations due to the tester moving inside the bus). The test conditions were realistic since the beacons were distant from each other and the bus was quite crowded.

We expected that, with the beacons securely installed in a higher location, the power values detected would have been significantly higher on the final installation compared to the average ones recorded in these tests. The motion activity detected between the check-in and check-out events (the two green vertical bars), as shown by the grey horizontal bar, is mostly “vehicle” (=3).

Figure 8b shows the received power values “Bus1” and “Bus2” for the switching from a bus to another scenario. The first red vertical line represents the first “enter event” in a beacon region. There is a short time interval (in yellow, called “waiting”) in which the trend of the received power from both sets of beacons starts from around −95 dBm, then tends to increase, since the tester was approaching the two buses. Immediately after, the received power values tend to split, indicating the tester boarded Bus2 (the first green line, i.e., the manual check-in on Bus2), and representing two separate trends, one averaging −70 dBm (for Bus2, the one boarded) and the other averaging −95 dBm (for Bus1, the more distant bus). 

When the tester alighted from Bus2 (second green vertical line, i.e., check-out Bus2) and boarded Bus1 (first purple vertical line, i.e., check-in Bus1), the two trends and their average values slowly switched. In this second phase, the average received power was −93 dBm for Bus2’s beacons and −67 dBm for Bus1’s beacons, up to the second vertical purple line (check-out Bus1). In the final section, the two points series both drop towards −95 dBm, corresponding to the user exiting from Bus1 and walking away from both vehicles. Since this test was carried out without the cars moving, no accelerometer related data is shown on the graph.

Figure 8c shows the power values from the sets of beacons positioned in the two buses for the Detecting beacons placed on two different buses scenarios. The orange dots (beacons on Bus2, on which the tester is traveling) show a stable trend for the whole trip, with an average value around −67 dBm, while the blue ones (beacons on Bus1) show an initially stable trend (Bus1 travels behind Bus2), then it starts to oscillate. 

This phenomenon is related to the change in position of Bus1 from behind Bus2 to its side, which corresponds to a slight increase in the received values of power emitted by the beacons placed on Bus1. Nonetheless, the trend of the values of Bus1’s beacons still show an average value of about −90 dBm, much lower than the power received from the beacons on Bus2 (average of −67 dBm). The horizontal grey activity bars indicate that, for most of the trip, the motion activity detection placed the smartphone on a “vehicle” (=3).

Figure 8d shows experimental data from the tests for the Waiting at a bus stop where a bus is idle scenario. After the enter event in the region defined by Bus1’s beacons, its received power values remain mostly below −80 dBm. When Bus2 arrives, the received power is initially at the same levels as Bus1’s beacons, but immediately after the tester starts the trip on Bus2 (first vertical green line, i.e., check-in Bus 2), the received power from its beacons settles around an average value of −73 dBm.

The power values from Bus1’s beacons drop again after the event represented by the second green vertical line (i.e., check-out Bus2), and even more after Bus2 leaves the “bus stop”, to eventually become non-existent when Bus2 leaves the range of the smartphone. At the same time, since Bus1 stayed at the bus stop, the received power from its beacon rises, but presents the same pattern as before Bus1’s check-in, with most values remaining below −80 dBm. No more values are recorded after the exit event (last vertical red line).

In the first part, between enter and Bus1’s check-in, the activity is mostly “walking” (=2), which is correct. However, during the bus ride, probably since the vehicle was not traveling fast enough (for safety measures) and the trip was very short, “vehicle” (=3) is only reported in the last half.

The few points shown in Figure 8e represent the detected power values emitted by the three beacons on the bus, which was passing close to the tester at a bus stop for the Waiting at a bus stop scenario. 

In this case the green vertical line corresponds to the instant when the bus stopped, but it can be immediately observed how the power never exceeds −85 dBm, even when the doors of the bus were open, and the user was close enough to them (less than 1 m). 

Also in this case, since the testers did not move from the bus stop, no data from the activity detection are represented in the graph. All of these tests helped greatly in reducing the range of available values to consider when setting a threshold for the minimum RSSI the system has detected to start the check-in process and a maximum for the check-out (Table 2). 

### 5.2. Setup for the Experiments on a Realistic Scenario

The vehicles available during the experimentation belonged to two categories based on their model: 10 of them were 12-m Citaro buses (owned by CTM SpA [32]), on each of which three beacons were installed; the other two were 30 m Skoda trams (owned by ARST SpA [36]), with seven beacons installed on each. 

Beacons were mounted close to the doors (as depicted in Figure 5) on the vehicles, behind some panels on the Citaro model, and inside some ceiling lights for the Skoda one. In both cases, the higher position relative to the users’ devices (which receive the signal) the number of beacons and their power are useful to reduce possible attenuations caused by the presence of people (human bodies which may cause interference, especially when the vehicle is crowded [37]) and of physical obstacles inside the vehicle.

The following values represent how we calibrated the parameters we used when setting the system:

- RSSI at 1m = −54 dBm. This parameter is useful for estimating the value for the proximity (one of “far”, “near”, or “immediate”, registered by the OS from every advertising packet right after receiving it).This value thus depends on the phenomena influencing Bluetooth signal propagation and reception (fading and other physical effects), such as reflections on the surfaces of the vehicle which cause reductions of the signal power received from users’ devices, as in the case when the vehicle presents several metal handles close to the beacons, as we encountered during some of our preliminary tests. The value chosen is the default one for the beacons we used.
- Identification codes:
  1. UUID = This parameter is identical for all of the beacons used during the experimentations and identifies a single beacon region which includes all PT providers and all vehicles in the system, and in particular all those affected by our tests.
  2. Major = integer identifying the vehicle in the system. It consists of a unique number associated with the vehicle number at a level of ticketing database/server. This allows to identify every vehicle by using the beacons installed onboard, whatever PT company they belong to (and whatever minor is assigned to them). Since the major is a 16-bit integer, using it this way allows us to identify 65.536 different vehicles in the area covered by the same UUID. For example, the Citaro vehicles used during the experimentation were associated to a major from 1 to 10, while the Skoda trams had 11 and 12.
  3. Minor = integer identifying the beacon in the vehicle. It is a unique number associated with a beacon inside a vehicle, and being also a 16-bit integer, it allows to identify 65.536 different beacons inside a vehicle identified by the same major (although unlikely). As an example, during our test, on the Citaro buses we had minors 1 to 3 (1 being the front, 2 the center, and 3 the rear) (Figure 5) and on the Skoda Vehicles minors 1 to 7.
- Password/security. All beacons in the system are accessible and configurable only by using a password and thus are protected from tampering by external attackers which might change the settings and make the system malfunction.

**Figure 9:**
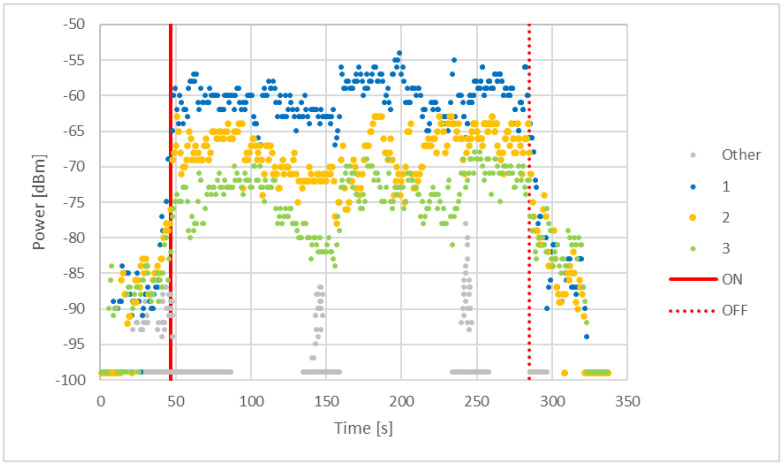
Power level inside a vehicle during the signal sampling tests.

Figure 9 shows the power levels for one of the tests we conducted in this last phase. The continuous red line represents the instant when testers boarded the vehicle, while the dashed red one corresponds to the alighting. Different colors are associated to the beacons (based on their *minor*) to differentiate them. The beacons numbered 1, 2 and 3 are the ones installed inside the vehicle, while the “Other” ones, whose power levels are represented by grey dots, are those installed in other vehicles nearby.

The graph highlights clearly how the power received from the “Other” beacons is rather distinct from the inside ones, with considerably lower values. The 3 beacons installed inside show similar patterns but almost parallel to each other, since the test was carried out on an almost fixed position inside the vehicle and the beacons were at different distances from the device. 

From the graph it is clear how beacon 1 was the closest one and beacon 3 was the one further away. The power levels from beacon 1 are all mostly over the Th_Pow_enter_bus threshold (−66 dBm, Table 2), meaning the check-in process could have started without issues (given all other checks were satisfied).

In this case once again the ±3 dB sensitivity for smartphones when calculating the value of a RSSI should be taken into account [35]. 

### 5.3. New Testing Scenarios

A series of new scenarios had to be defined, since it was important to test the automatic validation process not only when different vehicles were present nearby, but also taking into account the various possible circumstances when considering type and number of tickets, either already validated or not, possessed by a traveler. During the experimentation, to simplify the testing process, we decided to consider only two kinds of tickets, a 90-min ticket and a 120-min one, since the type of ticket was irrelevant to test the correct system operation in the different situations. We considered all of the scenarios found in Table 3. We remind that with the term “active ticket” we mean that the ticket has already been validated but it has not yet expired.

Most of these tests were executed using two devices (iPhone 6 and iPhone XS Max), and the vehicles were either traveling on-line or moving inside the depot. This means we could perform parallel tests and obtain more data, while also investigating the differences between an older device (iPhone 6, 2014) and a more recent one (XS Max, 2018). In fact, the latter showed a higher reactivity when detecting the beacons, since it uses different and newer technologies when compared to the iPhone 6 (in particular, the new motion sensors’ controller and library resolve several issues present in older iPhones, up to iPhone 6).

In June 2020 we made a first on-site survey to identify in which position the beacons would have to be installed inside the Citaro buses (CTM) and how many we needed to cover the whole length (12 m). As stated previously, 3 beacons were installed on each of the 10 vehicles available for testing, placed in a way which guaranteed a homogenous cover of the Bluetooth signal inside the vehicle. In July 2020 we performed some field tests when the beacon-equipped vehicles were on active duty, tracking them thanks to the information regarding their assignment to a specific bus-line and the relative expected passage times at specific stops. The tests carried out mid-July allowed us to identify and then resolve some bugs, which could only be experienced while the system was operating in the field.

Some of these tests were also performed on a vehicle traveling inside the bus-depot to increase the test execution frequency. In the following days, the data recorded during the tests was analyzed and some settings were modified accordingly, in particular some power thresholds used when detecting the enter and exit event for a beacon region (more precisely, enter_bus and exit_bus). In the meantime, more tests were carried on active-duty vehicles to check the new system performance after the changes. On 30 July, this testing session was concluded with a last batch of tests performed in the bus-depot. We preferred to continue using this method since it proved to be a good compromise, also considering how using public transport was still subject to the limitations imposed in Italy as a consequence of the COVID-19 pandemic. 

### 5.4. Test Results

Table 4 summarizes the results obtained during the last batch of tests. It is possible to notice how the previously fixed goals were fulfilled in most of the cases. At the app-level, users received the correct notification in all of the cases (check-in, transfer, multiple tickets, no tickets), and the results were also correct in the check-out scenario, for which we chose to show a notification at least for our internal tests in order to verify the performance of the system when detecting the end of the trip. From the server point of view, all validation events were correctly associated with the user account and the ticket which was actually used. In Table 4, the “Identifier” column encodes the type of test performed, and for every category shows the number of runs actually carried out (typically 10 or 20). We can show how, after resolving the bugs identified mid-July and having found the correct calibration, the success rate of the tests reached 100%, exception made for the cases where the motion activity check (Figure 2) was disabled to test if the system could work correctly even without this technology (and ultimately disproving this theory), as mentioned previously.

During the tests, we also verified the behavior of the system in the cases where two beacon-equipped side-by-side buses were present. The system was not hindered by these use conditions and actually proved to be reliable, since the validation happened correctly only on the vehicle the testers boarded, and not on the one close-by. This allowed us to conduct parallel tests, as if the other vehicle was not there.

From all of these tests we could then infer that the system is effective, detecting correctly the enter and exit of users from the vehicles (also different in type, size, operating speed, and provider in the cases of intermodality) and correctly executes validations in the server. Furthermore, the system correctly interfaces itself with the users, showing the correct notifications informing them of the occurred validation or asking them to perform some operations to allow the app to validate a ticket. A correct notification is also shown when the system informs users that, when they alighted the vehicle, their ticket was correctly updated to include information regarding the checkout event (which is not mandatory when performed manually).

## 6. Conclusions and Future Developments

The aim of our work was that of developing an innovative system for automating the process of ticket validation on public transport. The system we developed takes advantage of some of the hardware devices embedded on most smartphones, that are:

- Bluetooth, to receive and interpret the signals from BLE beacons, which are installed on the buses.
- GPS and positioning receivers, to identify users’ position and compare it to the coordinates of the closest bus stop and bus.
- Motion sensors, to detect how the users are moving, to know if they are riding on a vehicle.

These devices were used together to identify the patterns which allowed to clearly define if a person using the system was effectively traveling in a public transport vehicle or not. The system we tested was integrated in an already existing mobile ticketing application to simulate the operation of the finished product. We were able to test several different scenarios in different configurations of the system, mainly by having different vehicles in different positions between each other and compared to the testing devices. 

The results inferred from the data gathered during all of the tests, especially the ones which use the final calibration values for the parameters, show very promising achievements, with an almost perfect level of accuracy for the events in which the server updates the ticket information and at the same time those when the application shows the relevant notification to the users (validation, transfer, choosing a ticket, purchasing a ticket, check-out). The only exception to these results is actually due to our tampering, since we wanted to test if the system worked correctly when excluding the data from the motion sensors, but these tests ultimately failed, and we went back to the correctly working configuration.

While our internal tests, carried out with a limited number of devices, were a success, the next step would be to identify a larger sample of users, randomly selected from actual passengers, to perform a test on a larger scale. We would have liked to involve a larger sample during our tests, but we were limited by the restrictions dictated by the local government due to the COVID-19 pandemic.

Also, more buses would have to be equipped with BLE beacons to increase the chances of finding one traveling on the network and test the complete functionalities of the system. This will allow us to test the performance when several more devices are present in a vehicle and are communicating with the server. However, the system is configured in a way such as it will work correctly even when no beacons are detected, by using only location and motion sensors data. The solution we used was not the cheapest available one since the beacons we purchased were among the most powerful ones to allow us to test several configurations (and considering future studies unrelated to this project). Considering that, once a configuration setting is chosen, much cheaper devices can be selected, and considering they require very little maintenance, we could consider an estimated maximum cost of 20–30€ per vehicle (depending on the size). Other costs might be related to app development and server maintenance. Still, since these activities are already paid by the transport service companies for the mobile ticketing services, these would then be only incremental costs which may range from 1% to 5% of increase of the total cost.

Also, since we only had the iOS app available, we could not test the system on any Android device. We realize this is a significant limitation of our work, and we plan on conduct further testing with several different devices, specifically smartphones using Android OS. We plan on comparing the results from both iOS and Android devices, hopefully to show how our system works correctly regardless of the operative system installed. In fact, this issue has already been investigated, and it actually looks like a different OS does not impact negatively any result [38].

Another interesting feature we would like to implement is a gamification layer, which would assign a personal score based on the number of complete check-in/check-out cycles, performed both manually and automatically to also include those passengers who cannot use or prefer not to use the new system. We hope this system, when combined with the simplified validation process, would help to keep the users of the system interested in using it, and at the same time convince new people to start using it. At the same time, an advertising campaign will be used to inform all users about the new app and its updated functions, hopefully persuading even users who still prefer using traditional ticketing methods to “convert” to mobile ticketing.

## Figures and Tables

**Table 1:** System setting variables used during the experimentation.

	Pros	Cons
CI	CI is a legacy system available in every public transport service. It does not need necessarily any technology or connectivity since it can be implemented with simple paper tickets.	Passengers are required to check in manually when they enter each bus. Service providers may know when passengers enter the bus, but do not have information about when they alight.
CICO	CICO allows transport companies to monitor the flow of people, the permanence of each passenger in buses, and most important exchange nodes. Service providers can use the system to gather information about vehicle/line occupancy.	Passengers must manually check in when they enter the bus and check out manually when they exit the bus. The service provider needs to invest in more devices to be installed on their vehicles/stations.
CIBO	CIBO allows collecting the same kind of data as CICO systems, but passengers do not have to check out manually. Service providers can use the system to gather information about vehicle/line occupancy. Accuracy is high since check-out operations are automated.	Passengers must own a personal device (e.g., smart card, smartphone), buy virtual tickets, and bring this device with them. The service provider needs to invest in either smart cards or smartphone applications to allow for the additional functionalities.
BIBO	BIBO allows collecting the same kind of data as CICO and CIBO systems, but passengers do not need to interact with the system manually since the whole process is automated. Service providers can use the system to gather information about vehicle/line occupancy. Accuracy is very high thanks to the complete automation.	Passengers have to own a personal device (e.g., smart card, smartphone), buy virtual tickets, and bring this device with them. The service provider needs to invest in either smart cards or smartphone applications to allow for the additional functionalities. Automatic check-in should be thoroughly tested to avoid validating tickets while the user is not using public transport.

**Table 2:** System setting variables used during the experimentation.

Variable Name	Value
R_geofence	100 m
Enter_stop_radius	24 m
Exit_stop_radius	26 m
Th_Pow_enter_bus	66 dBm
Th_Pow_exit_bus	−99 dBm
T_stability	10 sec
R_beacon_region	approx. 15 m
Beacon Transmission Power	−4 dBm
Advertising interval	100 ms
RSSI at 1 m	−54 dBm (default)

**Table 3:** Test scenarios.

Identifier	Description	Prerequisites	Input	Objective
Boarding Case 1	A trip which starts with no active tickets.	Testers need to possess one or more tickets of the same type.	Testers have to wait at a bus stop/station and board the first incoming vehicle.	Testers should receive a validation notification within T-stability seconds and a check-in event should be created in the server.
Boarding Case 2	Boarding a vehicle shortly after a previous trip, with an active ticket.	Testers should have already completed (in order) Boarding Case 1 and Alighting Case 1.	Testers have to wait at a bus stop/station and board the first incoming vehicle.	Testers should receive a transfer notification within T-stability seconds and a check-in event should be created in the server.
Boarding Case 3	A trip which starts with no active tickets.	Testers need to possess many tickets of different types, and none should be already active.	Testers have to wait at a bus stop/station and board the first incoming vehicle.	Testers should receive a several tickets notification within T-stability seconds; they have to click on the notification, choose a ticket and validate it by simply touching a button; a check-in event should be created in the server.
Boarding Case 4	A generic trip which starts with no available tickets.	Testers need to not possess any valid ticket, neither active nor active.	Testers have to wait at a bus stop/station and board the first incoming vehicle.	Testers should receive a no tickets notification within T-stability seconds; they have to click on the notification, buy a ticket; within T-stability seconds, they should receive a Boarding Case 1 notification; a check-in event should be created in the server.
Boarding Case 5	A trip which starts at a bus terminal/station.	Testers need be at a bus terminal/station where many beacons equipped vehicles are idle and possess only no active tickets of the same type.	Testers have to wait at a bus terminal/station and board one of the idle vehicles.	Testers should receive a validation notification within T-stability seconds and a check-in event should be created in the server; the check-in event should be generated exclusively by the vehicle the tester boarded.
Alighting Case 1	The conclusion of a trip.	Testers should stay on a vehicle after completing Boarding Case 1.	Testers should stay on-board for ~5 min, then alight and leave the stop.	Testers should receive a check-out notification within T-stability seconds and a check-out event should be created in the server.
Alighting Case 2	The conclusion of a trip which ends at a bus terminal/station.	Testers should stay on a vehicle after completing Boarding Case 1.	Testers should stay on-board for ~5 min, then alight and stay at the stop/station.	Testers should receive a check-out notification within T-stability seconds and a check-out event should be created in the server.
Waiting	The testers wait at a bus stop/station without boarding any vehicle.	None	Testers should stay still while a beacon equipped vehicle transits close-by.	Testers should not receive any notification and no events should be recorded on the server.

**Table 4:** Main results of the different testing scenarios in terms of successful events.

Identifier	Date of the Test	N^r^ of Runs	Objectives	Successes
Boarding Case 1	29 July 2020	20	User notification (check-in)	100%
			Validation on the server	100%
Boarding Case 2	29 July 2020	10	User notification (transfer)	100%
			Validation on the server	100%
Boarding Case 3	29 July 2020	20	User notification (many tickets)	100%
			User notification (check-in)	100%
			Validation on the server	100%
Boarding Case 4	30 July 2020	20	User notification (no tickets)	100%
			User notification (check-in)	100%
			Validation on the server	100%
Boarding Case 5	29 July 2020	20	User notification (check-in)	100%
			Validation on the server	100%
Alighting Case 1	29 July 2020 30 July 2020	20	User notification (check-out)	100%
			Check-out on the server	100%
Alighting Case 2	29 July 2020	20	User notification (check-out)	100%
			Check-out on the server	100%
Waiting	16 July 2020 29 July 2020	10	No notification	50% *
			No validation on the server	50% *

* the last of these tests were performed ignoring the “motion activity” and this fact lead to the system not working properly.

## Data Availability

Data are not available due to legal and privacy issues.

## Appendix A

The following Table A1 gives the details available from the datasheet of the particular beacons we used during our experiments, available on the producer’s website [29].

**Table A1:** System setting variables used during the experimentation.

Configurable slots	Four slots are configurable for Eddystone frame types (UID, URL, TLM, EID) and the latter four slots are configurable for iBeacon, Quuppa, and Sensors frame types.
Radio characteristics	Version: Bluetooth 4.1, Low Energy (2.402 to 2.480 GHz) Module: Nordic SoC nRF51822 (MCU and transceiver) Antenna: printed meandered planar F-antenna
TX power	−40, −30, −20 to 4 dBm (with 4 dB steps). Programmable for each slot.
Power supply	Replaceable battery equal or equivalent to Panasonic CR2477 (typical capacity 1000 mAh @ 25 °C). The battery lifetime varies between 3 and 64 months, depending on the TX power and on the advertising interval
Other	Dimensions: 40 × 40 × 15 mm, for a weight of 20 g IP40 protection
